# Acetylation of HMGB1 by JNK1 Signaling Promotes LPS-Induced Peritoneal Mesothelial Cells Apoptosis

**DOI:** 10.1155/2018/2649585

**Published:** 2018-11-12

**Authors:** Shirong Cao, Shu Li, Yating Wang, Jiani Shen, Yi Zhou, Huiyan Li, Xueqing Yu, Haiping Mao

**Affiliations:** ^1^Department of Nephrology, The First Affiliated Hospital, Sun Yat-Sen University, China; ^2^NHC Key Laboratory of Nephrology, China; ^3^Guangdong Provincial Key Laboratory of Nephrology, Guangzhou 510080, China; ^4^Department of Nephrology, Central Municipal Hospital of Huizhou, Huizhou, China; ^5^Department of Rheumatology and Immunology, The Second Xiangya Hospital, Central South University, Changsha, China

## Abstract

Increased high mobility group box 1 (HMGB1) in dialysis effluence is associated with the presence of peritoneal dialysis-related peritonitis in patients and peritoneal dysfunction in acute peritonitis mice model, but it remains unclear whether HMGB1 is involved in peritoneal mesothelial cell injury and functions via molecular posttranslational modifications by acetylation in this process. Here we first showed correlation between HMGB1 acetylation level in dialysis effluence of patients and occurrence of Gram-negative peritonitis. The increased level of acetylated HMGB1 was similarly observed under the lipopolysaccharides (LPS) treatment in both human peritoneal mesothelial cell line (HMrSV5) and mice visceral peritoneum tissue. Overexpression of wild-type, but not hypoacetylation mutant of HMGB1, enhanced LPS-induced apoptosis in HMrSV5 cells, which was accompanied by elevated protein levels of BAX and cleaved-caspase 3 compared to the control. Pretreatment of HMrSV5 cell with JNK inhibitor attenuated LPS-induced HMGB1 acetylation. Consistently, primary peritoneal mesothelial cells from* Jnk1*^*-/-*^ mice showed a lower protein contents of acetylated HMGB1, fewer apoptosis, and decreased protein expression of BAX and cleaved-caspase3 after LPS exposure, as compared to those from wild-type mice. In conclusion, our data demonstrated HMGB1 promotes LPS-induced peritoneal mesothelial cells apoptosis, which is associated with JNK1-mediated upregulation of HMGB1 acetylation.

## 1. Introduction

Peritoneal dialysis- (PD-) related peritonitis remains the major clinical complication of peritoneal dialysis, resulting in marked morbidity, catheter loss, peritoneal dysfunction, and even death [1]. The peritoneum is primarily composed of an extensive monolayer of mesothelial cells and their soluble products [2]. Mesothelial cells are also participate in both sterile and infectious inflammation directly through processes such as autophagy [3] and indirectly through release of proinflammatory cytokines [4, 5]. For instance, our previous study showed that protein levels of HMGB1 were increased in the culture media of HMrSV5 treated with LPS [6].

HMGB1 is a nonhistone DNA-binding protein [7]. It localizes primarily to the nucleus of quiescent cells, but rapidly mobilizes to the cytoplasm and the extracellular space in response to exogenous and endogenous stimuli [8, 9]. When released from dead, injured, or immune cells, HMGB1 induces cytokine production, migration, proliferation, and differentiation by interaction with TLR2, TLR4, TLR9, and RAGE [10, 11]. Acetylation of specific lysine residues within the two nuclear localization sequences sites of HMGB1 has been suggested to regulate its intracellular shuttling in both immune and nonimmune cells [12, 13]. JNK signaling pathway regulates histone acetylation [14], and inhibitors of JNK have been shown to have anti-inflammatory effects in rheumatoid arthritis and other diseases [15]. However, the mechanism underlying this connection is still incompletely understood.

HMGB1 levels are enhanced in patients with strokes, acute myocardial infraction, and arthritis [16–18]. Notably, hyperacetylated HMGB1 in serum of patients is a definite biomarker to differentiate malignant mesothelioma patients from individuals occupationally exposed to asbestos and unexposed controls [19]. Our previous study revealed an elevated HMGB1 protein in the peritoneal dialysis effluence (PDE) of patients with peritonitis [6]. Treatment with HMGB1 antagonists ameliorates acute peritoneal inflammation and membrane dysfunction in the animal model of LPS-induced peritonitis [6]. Nevertheless, it remains unknown whether HMGB1 is acetylated and thereby promotes to peritoneal mesothelial cell injury during peritonitis.

In the present study, we assessed the protein levels of acetylated HMGB1 in the PDE of patients with peritonitis and determined the role of acetylated HMGB1 in LPS-induced mesothelial cells damage using HMrSV5, primary peritoneal mesothelial cells and acute peritonitis in mice. Further, we investigated the JNK1 signaling that regulates acetylation of HMGB1 in this process.

## 2. Materials and Methods

### 2.1. Collection of Peritoneal Dialysis Effluence

15 PD patients with Gram-negative peritonitis in our PD center were recruited based on the results of Gram stain and microbiological culture, and 19 PD patients without peritonitis were randomly selected served as controls. Two groups of patients were matched for age, sex, primary renal disease, duration of dialysis, and comorbidities. The PDE samples were collected as previously described [6]. This study was carried out with the approval of the Ethics Committee at the First Affiliated Hospital, Sun Yat-sen University (Guangzhou, China). All participants provided written informed consent.

### 2.2. Animals Study

Adult male C57BL/6J mice (20-25g) were from Guangdong Medical Experimental Animal Center (Guangzhou China).* Jnk1*^*-/-*^ mice (Strain name: B6.129S1-Mapk8^tmlFlv^/J; Stock number: 004319) were purchased from the Jackson Laboratory (Bar Harbor, Maine, USA). Acute peritonitis was induced in mice by intraperitoneal injection of a dose of 10 mg/kg of LPS (B4; Sigma-Aldrich, MO, USA) in 1 ml of sterile saline, as previously described [6]. Control mice received only sterile saline. The mice (n=6 each) were sacrificed at 48 h after injection. Visceral peritoneum was collected and frozen in liquid nitrogen. For the histological study, the tissue was fixed in formalin, dehydrated, and then paraffin embedded using standard techniques and subjected to the studies. All procedures were approved by the Animal Care and Use Committee of the Sun Yat-sen University.

### 2.3. Cell Culture, Establishment of Stable Cell Lines, and Treatments

Cell culture was performed as described previously [6]. HMrSV5 cells were a kind gift from Dr. Jian Yao (Department of Nephrology, Shanghai First People's Hospital, Shanghai, China). Mouse primary peritoneal mesothelial cells were prepared by intraperitoneal injection with a 0.25% trypsin/0.02% EDTA solution for 20 min. To harvest cells, cellular components were centrifuged at 1500 rpm for 10 min, washed with D-Hanks' balanced salt solution, and resuspended in Dulbecco's Modified Eagle's medium (DMEM)/F12 medium (Gibco, Thermo Fisher Scientific, Inc., Waltham, MA) supplemented with 15% fetal calf serum, 100 U/ml penicillin, and 100 g/ml streptomycin. Cells were seeded into 75cm^2^ plastic flasks and incubated at 37°C in a humidified 5% CO_2_ atmosphere. Cells were studied between passages 2 and 4.

Plasmid pEGFP-HMGB1 containing the full-length rat HMGB1 cDNA (Wt-HMGB1), the mutation of lysines 27-29 and 181-183 to arginines (mut-HMGB1), and empty vector pEGFP-N1 were from ForeverGen Co. Ltd. (Guangzhou, China). The HMrSV5 cells were transfected with wild-type or mutant HMGB1 constructs by Lipofectamine LTX and Plus reagent (Invitrogen Life Technologies, Paisley, United Kingdom). The empty vector pEGFP-N1 was used as a mock-transfection control. Transfected HMrSV5 cells were selected in medium that contained 200*μ*g/ml G418. Stable cell lines expressing Wt-HMGB1 and mut-HMGB1 fusion proteins were identified by immunoblotting with a monoclonal GFP antibody (Abcam, Cambridge, MA, USA). The stable transfected cell lines were cultured in DMEM/F12 medium supplemented with 10% fetal bovine serum, penicillin, streptomycin, and 200*μ*g/ml G418.

HMrSV5 or primary peritoneal mesothelial cells were cultured in serum-free medium for 24 h before treatment, and then incubated with 5*μ*g/ml LPS for different durations. Curcumin (Sigma, USA) was prepared in cell culture grade DMSO at a stock concentration of 1mM. HMrSV5 cells were pretreated with 1.25/2.5/5uM of curcumin for 4 h before LPS administration. Unstimulated controlled cells were incubated under serum-free conditions for either 12 h or 48 h, according to the needs of the experiment.

### 2.4. Histone Acetyltransferase (HAT) and Histone Deacetylase (HDAC) Activity Assay

After LPS treatment, nuclear extracts of HMrSV5 cells and primary peritoneal mesothelial cells from Wt and* Jnk1*^*-/-*^ mice were extracted using a Nuclear Extract Kit (Merck Milliporem, GER). Extracts were used to measured activities of both HAT and HDAC using Colorimetric Assay Kit (Biovision, USA) according to the manufacturer's instructions.

### 2.5. Coimmunoprecipitation Analysis and Western Blotting

Equal amount of proteins from cell or tissue extracts was precleared with protein A agarose beads (Pierce Biotechnology, Rockford, IL) and IgG isotype at 4°C for 1 h. After centrifugation, the supernatant was incubated with primary antibodies or normal rabbit serum as negative control overnight at 4°C with gentle shaking. The samples were processed as described previously [20].

PDE samples, cell culture media, whole-cell homogenate, or tissue lysates from the above studies were loaded onto a 10% or 15% polyacrylamide-SDS gel, electrotransferred onto a nitrocellulose membrane, blotted with the indicated antibodies, and then visualized by using an ECL western blotting detection kit (Amersham Bio-Sciences) according to the manufacturer's instructions. The following antibodies were used for the target proteins: anti-BAX antibody (Biosciences Pharmingen, San Jose, CA), anti-*β*-actin antibody (Boster Biological Technology. Wuhan, China), and anti-cleaved-caspase3 antibody (Cell Signal Technology. Beverly, MA, USA).

### 2.6. Immunofluorescence Staining

The peritoneal tissue sections were washed in phosphate-buffered saline, blocked with 5% bovine serum albumin in phosphate-buffered saline/0.3% Triton X-100 for 1h at room temperature, incubated with polyclonal rabbit anti-HMGB1 and anti-acetyl-lysine, polyclonal rabbit anti-acetyl-lysine, and monoclonal mouse anti-cytokeratin 18 (all antibodies from Abcam, Cambridge, MA, USA) at 4°C overnight, and followed by their corresponding secondary antibodies, including Alexa Fluro 546-conjugated anti-mouse IgG or Alexa Fluro 633-conjugated anti-mouse IgG (Cell Signal Technology. Beverly, MA, USA). Peritoneal mesothelial cells in peritoneum were identified by staining with monoclonal mouse anti-cytokeratin 18. The nuclei were counterstained with Hoechst 33258 (Sigma, USA). All images were captured by a confocal microscope (Zeiss LSM 510 META, Carl Zeiss, Oberkochen, Germany).

### 2.7. Apoptosis and DNA Content Assays

Apoptosis in cultured cells was assessed by flow cytometry using an Annexin V-FITC Apoptosis Detection Kit (Sigma, USA). In brief, cells were collected and resuspended in binding buffer. Annexin V-FITC and propidium iodide were added to produce a final concentration of propidium iodide of 1*μ*g/ml cell suspension and kept at room temperature for 15 min. Apoptotic cells were measured by flow cytometry. Apoptosis in histological sections was examined by terminal deoxynucleotidyl transferase-mediated dUTP nick end labeling (TUNEL) assay using an* in situ *cell death detection kit (Santa Cruz Biotechnology). Briefly, visceral peritoneum tissue sections were fixed with 4% paraformaldehyde and permeabilized with 0.2% Triton X-100. Fragmented DNA was labeled with fluorescein-12-dUTP at 37°C for 1 h. TUNEL-positive nuclei were detected with a fluorescent microscope. In transfected HMrSV5 cells expressing EGFP, due to the similar wavelength characteristics of Annexin V-FITC (excitation/emission: 494 nm/518 nm) and EGFP (excitation/emission: 488nm/509nm), it is not suitable to examine apoptotic transfected EGFP-HMrSV5 cells using Annexin V-FITC. Thus, apoptosis in EGFP-transfected HMrSV5 cells were analyzed by propidium iodide (PI; Sigma, P4170) staining, which was previously reported by other researchers [21, 22]. After LPS treatment, EGFP-transfected HMrSV5 cells were resuspended in PBS twice before fixation with 70% precooled ethanol. Prior to analysis, the cells were resuspended in PBS, stained with propidium iodide containing 50*μ*g/ml RNase A in the dark at room temperature for 30 min. The DNA content was analyzed by flow cytometry (Becton-Dickinson and Co., USA).

### 2.8. Statistical Analysis

The data are expressed as mean ± SE for at least three independent experiments. The difference between two groups was determined by unpaired student t test and among multiple groups was determined by one-way ANOVA. A p value less than 0.05 were considered significant. All statistical analyses were conducted using the standard statistical software SPSS 16.0 (SPSS Inc., Chicago, IL).

## 3. Results

### 3.1. HMGB1 Is Acetylated and Released in PD-Related and LPS-Associated Peritonitis

**Figure 1:**
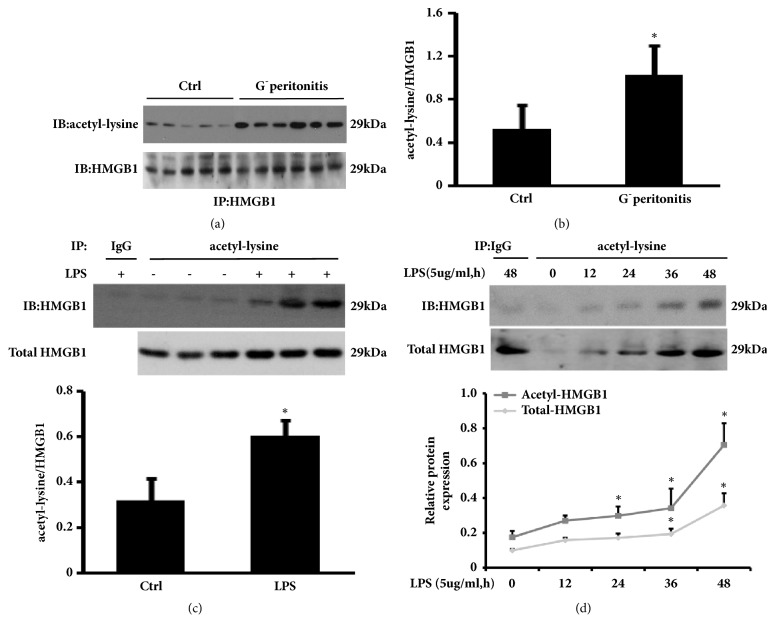
**Acetylated HMGB1 protein levels in PD patients and LPS-associated peritonitis.** (a) PDE samples of PD patients with Gram-negative peritonitis or without were pulled down with antibody against HMGB1 and the immunoprecipitation were probed with anti-acetyl-lysine, then stripped and reprobed for HMGB1. (b) Densitometry of acetyl-HMGB1 in immunoblots (relative to HMGB1). Data are means±SE (n=6). *∗*p<0.05* versus *control subjects. (c) Visceral peritoneum tissue lysates of control and LPS-treated mice were immunoprecipitated with anti-acetyl-lysine and immunoblotted for HMGB1. Densitometry of acetyl-HMGB1 normalized to HMGB1 in immunoblots. (d) HMrSV5 cell were treated with 5ug/ml of LPS for the indicated time period. Cell culture supernatants were immunoprecipitated with anti-acetylated lysine and probed with anti-HMGB1. The ratio of acetyl-HMGB1 to HMGB1 was quantitatively analyzed. Data in (c) and (d) are means±SE (n=6). *∗*p<0.05* versus* control group.

Our previous study showed that HMGB1 level in PDE is associated with presence of PD-related peritonitis [6]. It remains unclear whether the increased HMGB1 in PDE is acetylated during peritonitis. Therefore, we first ensured that HMGB1 was successfully pulled down from PDE and then probed with anti-acetylated lysine to identify the acetylation status of HMGB1. As shown in Figures 1(a) and 1(b), the levels of acetylated HMGB1 was significantly higher in dialysate from PD patients with peritonitis than those without peritonitis. These data suggest that HMGB1 of PDE is acetylated in patients with G^−^ peritonitis.

Given the link between acetylated HMGB1 and peritonitis, we sought to explore the contribution of peritoneal mesothelial cells in increased acetylated HMGB1 during PD-related peritonitis, using acute peritonitis mice model and HMrSV5 cells treated with LPS [6, 23]. On the basis of the findings in patients, it is reasonable that the visceral peritoneum tissue lysates from animal and cultured HMrSV5 cells were directly immunoprecipitated with anti-acetylated lysine and probed with anti-HMGB1. Our results showed that expression level of acetyl-HMGB1 was increased in visceral peritoneum tissue of LPS-associated peritonitis, compared to the control group (Figure 1(c)). In parallel, exposure HMrSV5 to LPS caused acetylated HMGB1 release to the cell culture supernatants in a time-dependent manner. Significant elevation in the level of total HMGB1 was detected at times as early as 24 h, with acetylated HMGB1 protein content lagging slightly behind (Figure 1(d)). These results revealed that peritoneal mesothelial cells might be, at least in part, responsible for enhanced acetylated HMGB1 in dialysate during PD-associated peritonitis. Unfortunately, the present study regarding peritoneal tissue and cultured HMrSV5 was not performed immunoprecipitation with anti-HMGB1 antibody to obtain an innately clearer acetylated HMGB1.

### 3.2. Hypoacetylation Mutant of HMGB1 Fails to Promote LPS-Induced Mesothelial Cells Apoptosis

**Figure 2:**
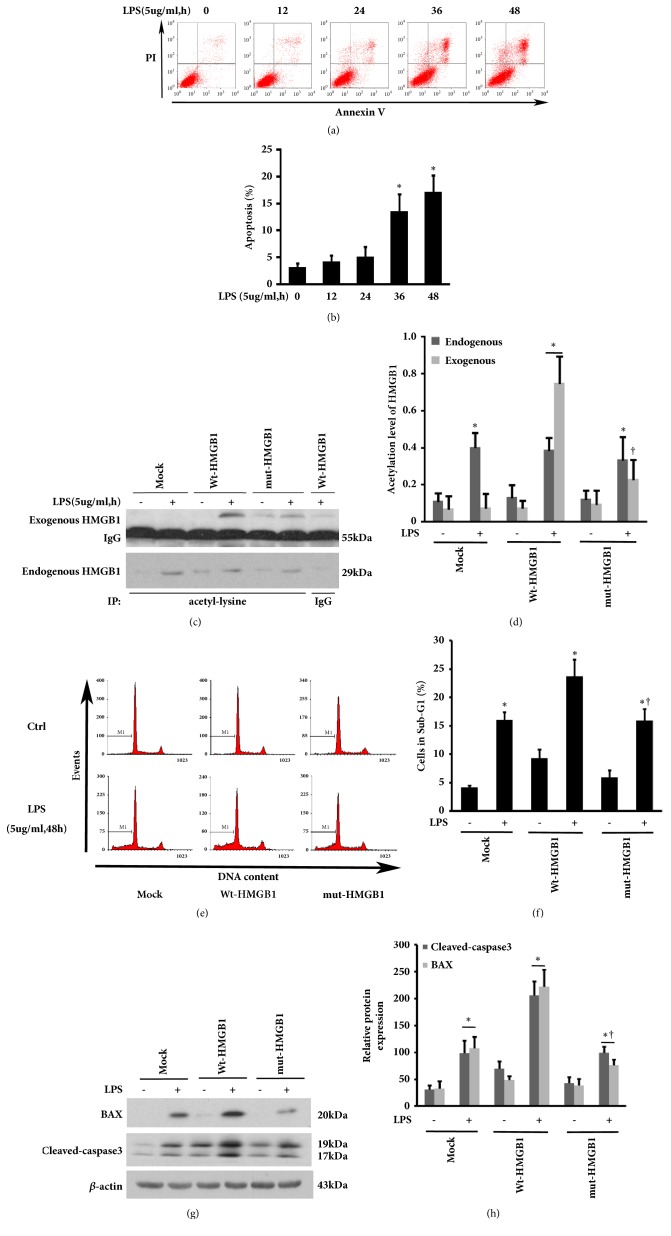
**Influence of Wt-HMGB1 and mut-HMGB1 on LPS-induced HMrSV5 cells apoptosis. **(a) Apoptosis in LPS-stimulated HMrSV5 cells was assessed by flow cytometry. (b) The apoptotic rate among different groups. Data are means±SE (n=3). *∗*p<0.05* versus *LPS-untreated cells. (c) HMrSV5 cells stably expressing Wt-HMGB1 or mut-HMGB1 were exposure to LPS (5ug/ml) for 48 hours. The empty vector pEGFP-N1 was used as a mock-transfection control. Exogenous and endogenous acetylated HMGB1 were examined by coimmunoprecipitation. (d) The acetylation levels of endogenous and exogenous HMGB1 were quantitatively analyzed by densitometer. (e) DNA contents were analyzed by flow cytometry. (f) The ratio of cells at sub-G1 stage. (g) The levels of BAX and cleaved-caspase3 were examined by immunoblotting assay. (h) Expression levels of the indicated proteins were quantitatively analyzed by densitometer and normalized with *β*-actin. Data in (d), (f) and (h) are means±SE (n=3). *∗*p<0.05* versus* same group without LPS exposure; †p<0.05* versus* Wt-HMGB1 transfected group with LPS treatment.

Pharmacological inhibition of HMGB1 has been shown to protect against LPS-induced acute peritoneal inflammation and dysfunction [6]. We reasoned that HMGB1 may play a role in LPS-mediated peritoneal mesothelial cells injury. As shown in Figures 2(a) and 2(b), LPS administration resulted in HMrSV5 apoptotic events in a time-dependent manner. It was reported that there were two frequently acetylated clusters of HMGB1, and the mutation of six lysines to arginines did not affect physiologic property of HMGB1, such as nuclear localization activity [14]. To further verify these frequently acetylated clusters of HMGB1 were required for mesothelial cells apoptosis by LPS treatment, the Wt-HMGB1 and lysine residues 27-29 and 181-183 mut-HMGB1 constructs were stably expressed in HMrSV5 cells. Upon LPS stimulation, an observed band appearing at 57-58kDa corresponded to the molecular weight of eGFP-HMGB1 (exogenous HMGB1) was pronounced in HMrSV5 cells transfected with Wt-HMGB1 as compared with the controls or mut-HMGB1 group. In contrast, there was no significant difference in acetylation of endogenous HMGB1 after LPS exposure among cells treated with mock, Wt-HMGB1, or mut-HMGB1 (Figures 2(c) and 2(d)). Additionally, flow cytometry analysis demonstrated that overexpression of wild-type, but not mut-HMGB1 caused a striking increase in the percentage of cells in sub-G1 stage after LPS stimulation from 16.08% to 23.80% as compared with empty vector (Figures 2(e) and 2(f)), which was accompanied by elevated protein levels of BAX and cleaved-caspase3 (Figures 2(g) and 2(h)). Notably, overexpression of either Wt- or mut-HMGB1 per se enhanced basal level of cleaved-caspase 3; the exact mechanism is unclear and needs to be determined. Collectively, these data suggested that acetylation in these two acetylated lysine clusters of HMGB1 is critical for LPS-mediated cell apoptosis.

### 3.3. HAT Inhibitor Curcumin Attenuates LPS-Induced Peritoneal Mesothelial Cells Apoptosis

**Figure 3:**
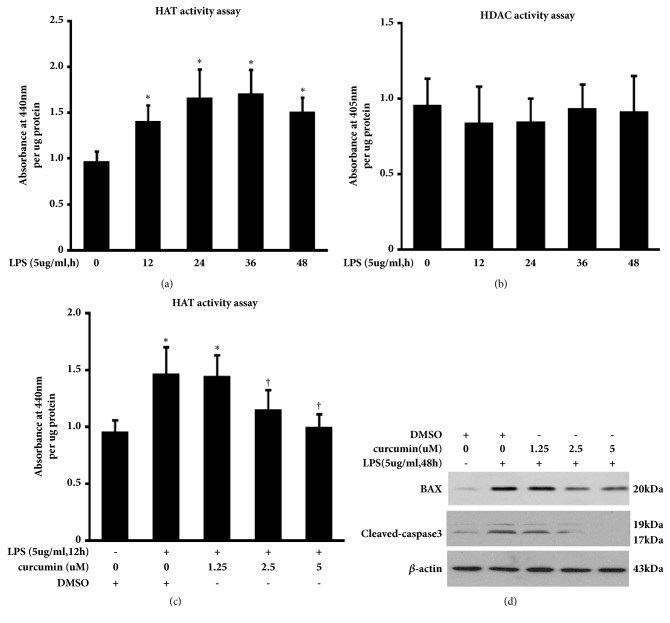
**HAT inhibitor curcumin reduces the level of BAX and cleaved-caspase3 in LPS-stimulated HMrSV5 cells. **(a) HAT activity in control and LPS-stimulated HMrSV5 cells was assessed by a Colorimetric Assay Kit as described in the Methods. Data are means±SE (n=3). *∗*p<0.05* versus *control group. (b) HDAC activity among different groups. Data are means±SE (n=3). (c) The effect of curcumin on HAT activity in LPS-treated HMrSV5 cells. Data are means±SE (n=3). *∗*p<0.05* versus *control group; †p<0.05* versus* cells with LPS-treated alone. (d) Expression levels of indicated proteins were determined by immunoblotting analysis.

Acetylation modification is the process regulated by the activities of both histone acetyltransferases (HAT) and histone deacetylase (HDAC). Nuclear HAT and HDAC activity in HMrSV5 were assessed by Colorimetric assays. We found that stimulation with LPS caused significant increases in the levels of nuclear HAT activity, but not HDAC activity in HMrSV5 (Figures 3(a) and 3(b)). Additionally, suppression of histone acetylation by HAT inhibitor curcumin blocked LPS-induced HAT activity (Figure 3(c)) and expression of BAX and cleaved-caspase3 (Figure 3(d)). The expression of BAX and cleaved-caspase 3 was also observed in cells treated with DMSO alone that might be due to the effect of DMSO in cellular function like lipid metabolism and apoptosis [24, 25]. These data suggest that the HAT inhibitor, curcumin, may alleviate LPS-induced peritoneal mesothelial cells apoptosis.

### 3.4. JNK1 Inactivation Reduces HMGB1 Acetylation in LPS-Treated Peritoneal Cells

**Figure 4:**
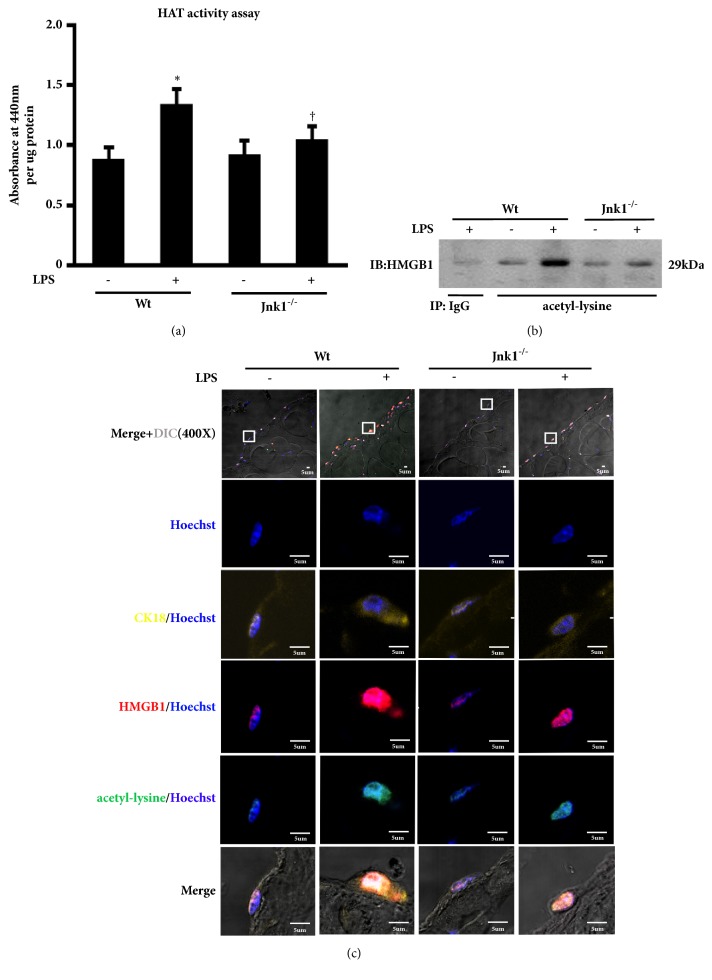
**The role of JNK signal activation in LPS-induced HMGB1 acetylation.** (a) Primary peritoneal mesothelial cells from wild-type (Wt) and* Jnk1*^*-/-*^ mice were treated with LPS for 48 h, and then examined HAT activity. (b) Cells were treated and described as above. Acetyl-HMGB1 level was examined by immunoprecipitation. (c) Parietal peritoneum in each group was stained for CK18 (yellow), HMGB1 (red), and acetyl-lysine (green). Hoechst (blue) was used for nuclear staining. Scale bar: 5um. Inserts showed particular area at higher magnification to better visualize the location of HMGB1 and acetyl-lysine.

JNK1 activation was observed in LPS-associated peritonitis and LPS-treated HMrSV5 cells in our previous study [3]. Other researchers found that JNK phosphorylation mediated histone acetylation by increasing HAT activity or inhibition of SIRT1 activity [26, 27]. Thus, we sought to investigate whether JNK activation is involved in LPS-induced HMGB1 acetylation. We found that only JNK inhibitor SP600125 reduced acetylation level of HMGB1 after LPS stimulation, whereas this response was not attenuated by pretreatment with ERK inhibitor PD98059 and p38 inhibitor SB203580 (data not shown). The findings indicate that suppression of JNK was associated with reduced acetylation of HMGB1 in this model. To further confirm the role of JNK signaling upon LPS-evoked HMGB1 acetylation, we isolated and cultured primary peritoneal mesothelial cells from Wt and* Jnk1*^*-/-*^ mice. Consistently, the LPS-mediated increasing of HAT activity and acetylated HMGB1 was reduced in mesothelial cells from* Jnk1*^*-/-*^ as compared with those from Wt mice (Figures 4(a) and 4(b)). Moreover, immunofluorescence staining showed that HMGB1 was primarily located in the nucleus of LPS-untreated mesothelial cells from Wt mice, and LPS treatment increased the content of HMGB1, with most acetylated HMGB1 translocated from the nucleus to the cytoplasm. By contrast,* Jnk1*^*-/-*^ mesothelial cells manifested reduced HMGB1 in both nucleus and cytoplasm after LPS administration (Figure 4(c)). These data suggested that JNK1 activation is associated with LPS-induced HMGB1 acetylation in peritoneal mesothelial cells.

### 3.5. JNK1 Deficiency Attenuates LPS-Induced Peritoneal Mesothelial Cells Apoptosis

**Figure 5:**
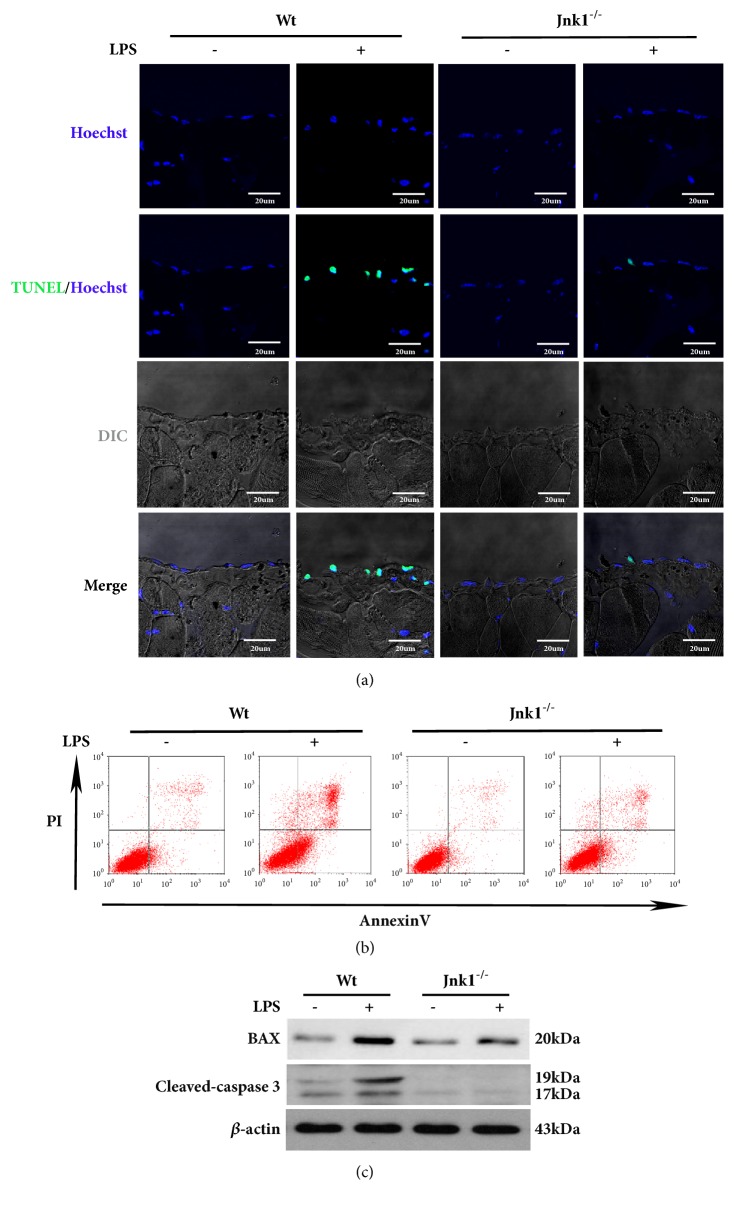
**JNK1 knockout decreases peritoneal mesothelial cells apoptosis induced by LPS. **(a) Representative images of TUNEL staining in parietal peritoneum of Wt and* Jnk1*^*-/-*^ mice after LPS treatment. Scale bar: 20um. (b) Peritoneal mesothelial cells derived from Wt or* Jnk1*^*-/-*^ mice and exposed to LPS, cell apoptosis was examined by flow cytometry. (c) Cell lysates were probed with antibodies against BAX and cleaved-caspase 3.

Since inhibition of HMGB1 acetylation suppresses cell apoptosis, we then examined whether JNK1 functions upstream of HMGB1 acetylation to promote LPS-induced cell apoptosis. We found that there were fewer TUNEL-positive apoptotic mesothelial cells in the peritoneum of* Jnk1*^*-/-*^ than those in Wt mice following LPS treatment (Figure 5(a)). Consistently, compared to peritoneal mesothelial cells derived from wild-type mice, those from* Jnk1*^*-/-*^ mice showed a significant reduction in the rate of apoptotic cells after LPS exposure (Figure 5(b)). Further, the expression of BAX and cleaved-caspase3 was drastically decreased in LPS-treated* Jnk1*^*-/-*^ cells (Figure 5(c)). Collectively, these data demonstrated that JNK1 play an essential role in LPS-induced apoptosis in peritoneal mesothelial cells.

## 4. Discussion

The present study demonstrates elevated acetylation of HMGB1 protein in PDE of patients with peritonitis and its impact in peritoneal mesothelial cell apoptosis. By stably expressing wild-type HMGB1 or hypoacetylation mutant HMGB1 in HMrSV5 cells, we showed that overexpressed wild-type, but not mutant HMGB1, increased LPS-induced apoptosis. Pretreatment with the JNK inhibitor or ablation of JNK1 gene in mesothelial cells attenuated LPS-triggered HMGB1 acetylation, expression of BAX and cleaved-caspase3, and apoptosis. Our findings suggested that LPS-induced HMGB1 acetylation via JNK1 signaling may be an important determinant of apoptosis regulation in peritoneal mesothelial cells.

HMGB1 is recognized as a proinflammatory cytokine that plays a significant role in the pathogenesis of many diseases, especially inflammation disease and cancer [28, 29]. Some investigations have demonstrated that serum/plasma HMGB1 concentrations are elevated in patients with strokes, acute myocardial infarction, and rheumatic arthritis [30]. Others found that both patients with malignant mesothelioma and healthy population exposed to asbestos had higher HMGB1 expression in the serum, but HMGB1 only in patients with malignant mesothelioma are highly acetylated [31]. Furthermore, hyperacetylated HMGB1 is more specific and sensitive serum biomarker to diagnosis of malignant mesothelioma than HMGB1 [19]. We previous reported that HMGB1 levels in PDE were higher in patients with peritonitis than those in controls, and gradually declined during the period of effective antibiotic treatments [6]. However, it is unclear whether the increased HMGB1 in PDE is acetylated. In the present study, we confirmed that acetylated HMGB1 in PDE of patients with clinical peritonitis were significantly increased compared to those in control subjects. In addition, the increased expression of acetylated HMGB1 was similarly observed under LPS treatment in both human peritoneal mesothelial cell line and mice visceral peritoneum tissue. All these findings intrigued us to speculate that the pathogenic effect of HMGB1 might contribute to its acetylation in PD-related peritonitis.

Posttranslational modification of HMGB1 appears to play a critical role in mediating cell injury [32]. Accumulating evidence has shown that histone deacetylase inhibitors have anti-inflammation and protective effects in some disease models or pathological conditions, including ischemia reperfusion-mediated damage, tumor cell proliferation, and fibrosis [33, 34]. But it has not yet been determined that HMGB1 can be acetylated in LPS-induced peritoneal mesothelial cells. Here, we showed that LPS treatment led to increased protein level of acetylated HMGB1, concomitant with elevation in peritoneal mesothelial cell apoptosis, in both acute peritonitis mice model and HMrSV5 cells stimulated by LPS. Double mutants of acetylated clusters of HMGB1 have been revealed nonresponse to TSA stimulation and failed to translocate to the cytoplasm [12], even though it is unclear whether the multiple mutations change the protein's folding and other function. In line with previous studies, through the site-directed mutagenesis on lysines in the nuclear localization sequences region, we found that overexpressed wild-type but not mutant HMGB1 enhanced LPS-induced BAX and cleaved-caspase 3 expression, as well as apoptosis, when compared with empty vector. Collectively, our findings suggest that acetylation of HMGB1 is critical for LPS-mediated cell injury. However, whether acetylation of HMGB1 is directly capable of inducing cell apoptosis in vivo and in vitro remains elusive, and it is worthwhile to investigate further in the future.

Compelling evidence from many laboratories indicates that JNK activation mediates various physiological processes, including inflammation, cell proliferation, survival, and apoptosis in response to diverse events. Blockade of the JNK pathway by pharmacological (e.g., SP600125) or genetic means (transfection with JNK1 siRNA) leads to a reduction of histone acetylation [35, 36]. In vitro experiments showed that enhancing phospho-JNK1 activity was correlated with an elevated acetylated H3 histone in trigeminal ganglion neurons, and H3 acetylation was attenuated by SP600125 [37]. Additionally, sustained JNK1 activation was also found to be associated with an upregulation of histone H3 methylation in human hepatocellular carcinoma tissues [38]. Thus, the JNK signaling may affect the posttranslational modifications of proteins. In this study, we found that the JNK inhibitor, SP600125, reduced acetylation level of HMGB1 in HMrSV5 cells after LPS treatment. Further, the activity of HAT and expression of acetylated HMGB1 was lower in primary peritoneal mesothelial cells from* Jnk1*^*-/-*^ than those from Wt mice after LPS exposure. Concurrently, the expression of BAX and cleaved-caspase3 was dramatically decreased in LPS-treated* Jnk1*^*-/-*^ cells. Immunofluorescence staining of peritoneum revealed that* Jnk1*^*-/-*^ mesothelial cells manifested decreased HMGB1 in both nucleus and cytoplasm, less apoptotic cells following LPS administration. These observations suggest that JNK1 promote LPS-induced apoptosis in peritoneal mesothelial cells, which is associated with JNK1-mediated upregulation of HAT activation and subsequent HMGB1 acetylation.

## 5. Conclusions

In summary, the present study suggests that elevated acetylation of HMGB1 in PD-associated peritonitis may promote peritoneal mesothelial cell apoptosis. Regulation of HMGB1 acetylation by suppressing JNK1 activity represents a strategy for protection against LPS-induced injury in peritoneal mesothelial cells and has profound implications for the treatment of human PD-related peritonitis.

## Data Availability

The data used to support the findings of this study are available from the corresponding author upon request.
